# Memory inflation: Beyond the acute phase of viral infection

**DOI:** 10.1111/cpr.13705

**Published:** 2024-07-11

**Authors:** Yanfei Li, Jie Xiao, Chen Li, Mu Yang

**Affiliations:** ^1^ School of Basic Medical Sciences Chengdu University of Traditional Chinese Medicine Chengdu China; ^2^ Centre for Translational Research in Cancer, Sichuan Cancer Hospital and Institute, Sichuan Cancer Center, School of Medicine University of Electronic Science and Technology of China Chengdu China

## Abstract

Memory inflation is confirmed as the most commonly dysregulation of host immunity with antigen‐independent manner in mammals after viral infection. By generating large numbers of effector/memory and terminal differentiated effector memory CD8^+^ T cells with diminished naïve subsets, memory inflation is believed to play critical roles in connecting the viral infection and the onset of multiple diseases. Here, we reviewed the current understanding of memory inflated CD8^+^ T cells in their distinct phenotypic features that different from exhausted subsets; the intrinsic and extrinsic roles in regulating the formation of memory inflation; and the key proteins in maintaining the expansion and proliferation of inflationary populations. More importantly, based on the evidences from both clinic and animal models, we summarized the potential mechanisms of memory inflation to trigger autoimmune neuropathies, such as Guillain‐Barré syndrome and multiple sclerosis; the correlations of memory inflation between tumorigenesis and resistance of tumour immunotherapies; as well as the effects of memory inflation to facilitate vascular disease progression. To sum up, better understanding of memory inflation could provide us an opportunity to beyond the acute phase of viral infection, and shed a light on the long‐term influences of CD8^+^ T cell heterogeneity in dampen host immune homeostasis.

## Graphical abstract

This review highlights the definition of virus‐induced CD8^+^ T cell memory inflation，as well as the impacts on multi‐diseases progression. By summarizing current understandings, an opportunity is provided for shedding a light on the long‐term influences of memory inflation in host‐immune homeostasis thus beyond the acute phase of infection.

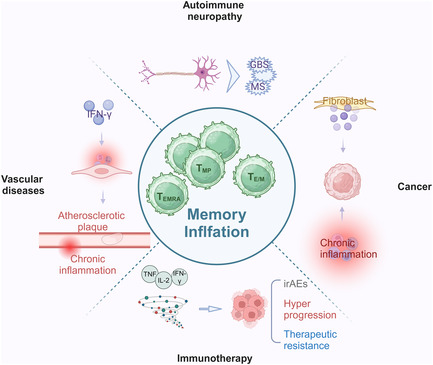

## 1 INTRODUCTION

The global outbreak of COVID‐19, caused by novel coronavirus, has affected people worldwide.^1^ Other than a significant portion suffering from systemic inflammation and respiratory failure during the acute phase, epidemiological studies have proved that majority of patients only experienced mild symptoms or even showed asymptomatic throughout the infection.^2^, ^3^ However, in neglecting the severity of their symptoms at acute phase, the whole infected population were reported to obtain alternative host immune backgrounds after recovery, especially the dynamic changes of CD8^+^ T cell subsets, functions, and responsiveness.^4^ Preciously studies have already documented that the most prominent characteristic of CD8^+^ T cells in post‐infection is the high turnover rate of memory phenotype without virus antigen participation.^5^, ^6^, ^7^ Unfortunately, few evidences emerged to reveal the associations between this immune heterogeneity with any clinical manifestations, until immune abnormalities‐mediated diseases are rising in recovered patients of COVID‐19 infection, such as idiopathic interstitial pneumonia, autoimmune neuropathies and tumorigenesis.^8^ Therefore, growing attentions are paid to the post‐infection induced dysregulation of CD8^+^ T cells, which might be one of the decisive triggers for such immunological disorders, and/or bring poor prognosis to patients.^9^, ^10^

Apart from COVID‐19, cytomegalovirus (CMV) infection is the most potent factor in mediating similar immunophenotypes of CD8^+^ T cells. Different from post‐infection of lymphocytic choriomeningitis virus (LCMV), which primarily leads to exhaustion phenotypes of CD8^+^ T cells for resisting viral colonization in somatic cells, CMV provokes CD8^+^ T cells after virus clearance due to immunological memory.^11^ The expansion of CD8^+^ T cells by CMV is confirmed to produce large number of memory‐like T cells, including memory precursor (MP), effector/memory (E/M), as well as terminal differentiated effector memory (EMRA) subsets with antigen‐independent manner in both animal models and human population.^12^, ^13^, ^14^ This exclusive phenotype known as “memory inflation,” is also commonly observed after vaccination, and described as a life‐long “population expansion” of CD8^+^ memory T cells in circulating system.^11^, ^15^ Notably, similar with COVID‐19 infection, recent reports revealed that excessive differentiation of memory‐inflated CD8^+^ T cells after CMV infection exhibits a close relationship with both autoimmunity and tumorigenesis.^16^, ^17^, ^18^, ^19^

The latest research studies have largely explored the mechanisms underlying memory‐ inflated CD8^+^ T cells, including their characteristics, key molecules involved in this unique phenotypic differentiation and expansion, as well as the alternative immune functions of inflationary CD8^+^ T cells.^20^, ^21^, ^22^, ^23^ Thus, to better understand the potential roles of memory inflation in mediating disease onset, progression, and side effects during treatment, we in‐depth review the current knowledge in the molecular features of memory‐inflated CD8^+^ T cells, along with the differentiation patterns. Additionally, the critical roles of CD8^+^ T cells with memory inflation phenotype in autoimmune diseases of nervous system, treatment resistance of cancer immunotherapies, and vascular disorders would be also addressed.

## 2 DEFINITION OF MEMORY INFLATION

### 2.1 Basic concept of memory inflation

The concept of memory inflation was firstly introduced according to the report of continuous accumulations of viral antigen specific CD8^+^ T cells in 2003.^24^ By using murine CMV (MCMV), a C57BL/6‐based murine model was generated to investigate the significant accumulation of MCMV IE1 protein (pp89, also known as m123/phosphoprotein)‐specific CD8^+^ T cells.^25^ After 1 year post‐infection, inflated populations could still be observed in both circulating system and multi‐organs.^25^ This phenomenon, termed memory inflation, was hypothesized to begin shortly after primary infection and persist a long period without viral‐antigen participation.^24^ Subsequent studies confirmed that CMV‐mediated cellular immunity in patients could initiate a similar memory inflation of CD8^+^ T cells, and further investigations also revealed a long‐term expansion of antigen‐specific CD8^+^ T cells in the context of vaccination.^25^, ^26^ Interestingly, other than CMV, similar inflationary CD8^+^ T cells could be also induced by viruses that do not establish latency, such as replication‐defective adenovirus, polyomavirus, and parvovirus.^27^, ^28^ However, CMV remains the most efficient trigger in bringing memory inflation of both human and rodent models.^28^

**FIGURE 1:**
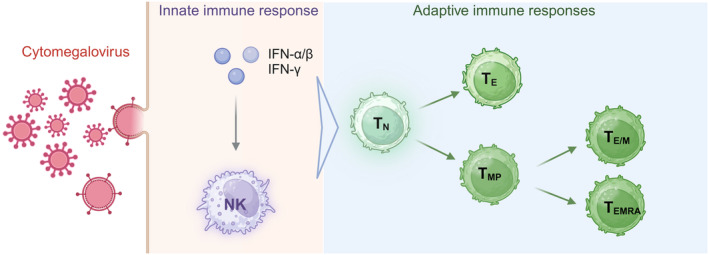
The host's immune responses to CMV infection. Upon viral infection, IFN‐α/β and IFNγ activated NK cells to provide an early antiviral response, followed by clonal expansion and differentiation of CD8^+^ T_N_ cells into T_E_. And then a small amount of primed CD8^+^ T cells give rise to phenotypically diverse CD8^+^ T_MP_ cells, which expanded and eventually stabilized as CD8^+^ T_E/M_ and T_EMRA_ cells. NK, Natural Killer; T_N_, Naïve T cells; T_E_, Effector T cells; T_MP_, Memory precursor T cells; T_E/M_, Effector/Memory T cells; T_EMRA_, terminally differentiated effector memory T cells.

The initial priming of CMV infection involves a hierarchical and redundant process, with the involvements of type I and II interferons (IFN‐α/β and IFNγ), natural killer (NK) cells from innate immune system, and CD4^+^ and CD8^+^ T cells that belong to adaptive immunity.^29^ Upon viral infection, IFN‐α/β and IFNγ activated NK cells to provide early antiviral responses, followed by clonal expansion and differentiation of naïve (N) CD8^+^ T cells into effector (E) populations on basis of adaptive immune responses against viral infection.^30^ Subsequently, CD8^+^ T_E_ cells are responsible for controlling infection and reactivating to latent infection until eliminating the viral titers.^24^, ^31^ At the same time, a small amount of primed CD8^+^ T cells give rise to phenotypically diverse T_MP_ subsets that are categorized by their homing property, cytokine polyfunctionality, and cytolytic capacity.^32^, ^33^ Due to continuous exposure to latently expressed antigens, expansion and asymmetric differentiation are initiated in those T_MP_ cells that specifically recognized viral antigens.^21^ As a result, high frequencies of memory‐like CD8^+^ T cells expanded and eventually stabilized with distinct profiles, such as CD8^+^ T_E/M_ and T_EMRA_ cells (Figure 1).^34^, ^35^, ^36^ In this process, both percentile and absolute numbers of CD8^+^ T_N_ cells were monitored to constantly decline.^13^, ^37^ Longitudinal study on CMV infection spanning 27 years also mentioned that CMV seropositive individuals have significantly higher numbers of CD8^+^ T_E/M_ and T_EMRA_ cells, whereas the percentile of CD8^+^ T_N_ cells and T central memory (CM) populations remain decreasing.^38^ In fact, compared to non‐inflated T cells, inflated T cells present at high frequencies in both blood and tissues, such as liver and lung.^39^ And the average fraction of CMV‐specific cells within the general memory compartments may exceed 10% in inflated status.^40^ Therefore, the immunological aspect of memory inflation could be generally defined as redundant CD8^+^ T_E/M_ cells with diminished CD8^+^ T_N_ cells in circulating system.^27^, ^41^, ^42^, ^43^

### 2.2 Phenotypic features of memory inflated CD8 ^+^ T_E_ _/M_ cells

Virus‐specific memory CD8^+^ T cells could be mainly categorized into three populations based on their phenotypic features and tissue distributions: T_CM_, T_E/M_, and T_EMRA_ cells, whereas CD8^+^ T_E/M_ cells are considered as the predominant population among them.^35^, ^44^ Accordingly, T_CM_ cells possess homing property and primarily reside in secondary lymphoid organs, along with excellent sensitivity to the proliferative cytokines interleukin‐7 (IL‐7) and interleukin‐15 (IL‐15).^42^, ^45^, ^46^ Besides, T_E/M_ and T_EMRA_ cells exhibit a terminally differentiated phenotype with cytolytic functions, are predominantly found in circulating system, but often patrolling at the site of peripheral organs with primary infection.^41^, ^42^, ^43^, ^46^ Actually, on account of the sophisticated trajectories among T_CM_, T_E/M_ and T_EMRA_ cells under certain conditions, various markers are currently used to distinguish T_E/M_ cells from inflationary population.^26^ In general, CD8^+^ T cells under memory inflation display a highly differentiated phenotype, which are characterized by reductions of homing markers (CD62L and CCR7).^43^ As the sign of T_CM_ cells, IL15Rβ (CD122) is also downregulated in CD8^+^ T_E/M_ cells.^47^, ^48^ Meanwhile, T cell Ig and ITIM domain (TIGIT) expression is found to decrease in CD8^+^ T_E/M_ cells for retaining the secretion of Grazyme B and perforin.^49^ And low level of IL7Rα (CD127) expression in primed CD8^+^ T cells implies the transition of T_MP_ to T_E/M_ and T_EMRA_ phenotypes.^50^ On the other hand, significant elevating of CD25 and CD69 expressions were observed in CD8^+^ T_E/M_ cells as well.^51^ But consistently expression pattern of killer cell lectin‐like receptor G1 (KLRG1) exerts inhibitory effects on CD8^+^ T_E/M_ cells for preventing excessive activation.^52^ In murine models of memory inflation, stemness marker CD44 is detected to constitutively express from the generation of CD8^+^ T_MP_ cells to CD8^+^ T_E/M_ population formation.^53^ Moreover, CMV‐specific CD8^+^ T_E/M_ cells from patients own high expressions of CD45RO during primary infection, but sequentially enrich CD45RA upon the chronic phase.^54^ According to the alternative immune functions of CD45 isoforms, co‐stimulatory signals were confirmed to be needed for the maintenance of CD8^+^ T_E/M_ population, which bring the expressions of co‐receptors (CD27 and CD28).^55^, ^56^ Recent studies identified that asymmetric differentiation could cause certain amount of CD8^+^ T_E/M_ cells express the replicative senescence markers, such as CD57 and CD85 to further shape the CD8^+^ T_EMRA_ population.^57^, ^58^ Simultaneously, expanded CD8^+^ T_E/M_ population was detected to possess high expression of CX_3_C‐chemokine receptor 1 (CX_3_CR1) that allows them to interact with endothelial cells and receive survival signals from antiapoptotic protein BCL‐XL.^11^ Collectively, co‐expression of CD25, CD69, KLRG1, CD27, CD28 and CD45RO (CD44 for murine model) in combine with the reduction of TIGIT, CD127, CD122, CD62L, and CCR7 in CD8^+^ T cells is used as current strategy to categorize CD8^+^ T_E/M_ cells, whereas expressions of CX_3_CR1, CD57 and CD85 are usually to further dissect the T_EMRA_ cells from CD8^+^ T_E/M_ subset, or distinguish CD8^+^ T_E_ cells in recall responses that mediated by CD8^+^ T_CM_ and T_E/M_ cells (Table 1).^59^

**TABLE 1:** Characteristics of Virus‐specific CD8^+^ T cells.

	Central memory	Inflationary	Exhausted
Homeostatic proliferation	++	−	−
Antigen dependence	−	++	++
Cytokine polyfunctionality	++	+ (low% IL‐2)	−
*Homing markers*			
CD62L	++	−	−
CCR7	++	−	−
CX_3_CR1	−	+/−	
CD44		++	
*Cytokine receptors*			
CD122	++	−	
CD127	++	+/−	
*Natural killer receptors*			
KLRG1	−	++	−
CD57	−	+/−	
CD85	−	+/−	
*Co‐stimulatory receptors*			
CD28	+	−	−
CD27	++	−	−
*Inhibitory receptors*			
PD‐1/CTLA‐4/LAG3/TIM3/ etc.	−	−	++
*Transcription factors*			
T‐BET	−	+	+/−
EOMES	−	+/−	+

*Note*: − absent or low, +/− intermediate, + high, ++ prominent.

Irrespective of their dynamic expressions of surface markers, CD8^+^ T cells initially express Granzyme B and perforin at acute phase of CMV infection, and then efficiently produce high levels of IFNγ and tumour necrosis factor (TNF) during the latent phase.^26^, ^60^ Accordingly, CD8^+^ T_EMRA_ cells appear at the latent phase of infection have more robust ability to secret IFNγ and TNF.^61^ Interestingly, except of the secondary responses, Granzyme B and perforin are almost completely absent at the latent phase.^26^ In contrast, investigations from post‐infected patients indicated that circulating CD8^+^ T_E/M_ cells emerge the ability to express Granzyme B and perforin, although the secreting levels vary depend on the heterogeneities of individuals.^62^, ^63^ Furthermore, TNF level of CD8^+^ T_E/M_ cells is found to decrease in infected patients by comparing with the same individuals that were experiencing the acute phase of infection.^64^ It seems that human‐inflated CD8^+^ T cells are more likely to perform cytolytic abilities, whereas murine CD8^+^ T_E/M_ cells from memory inflation might generally mediate a widespread immune response via inflammatory cytokines releasing.^65^, ^66^ And according to the differences between human population and laboratory rodent in contacting various pathogens, environmental diversity might be another factor to prompted this controversial aspect of functional cytokines secreting from CD8^+^ T_E/M_ cells.^67^, ^68^ It is also worth mention that modest amount of interleukin‐2 (IL‐2) is detected from CD8^+^ T_E/M_ cells in patients at the acute phase, as well as virus‐specific CD8^+^ T_E/M_ cells from infected mice in both acute and chronic phases.^11^, ^69^ But viral‐antigen nonspecific murine CD8^+^ T_E/M_ cells with chronic infection could produce excessive IL‐2.^70^ And recent studies have shown that autocrine IL‐2 programs the optimal secondary expansion of memory CD8^+^ T population in an acute infectious model due to the critical role of IL‐2 in deriving T cell priming.^28^ Thus, we speculated that memory inflation derived CD8^+^ T_E/M_ cells still exhibit self‐renewal ability via IL‐2 signalling pathway after viral clearance. Overall, other than surface markers, constitutively secreting of IFNγ, TNF and IL‐2 could be considered as the alternative characterization of memory inflated CD8^+^ T cells.

### 2.3 Inflation and exhaustion of CD8 ^+^ T cells after viral infection

Chronic viral infections involve a process of viral adaptation to the host, leading to various outcomes.^71^, ^72^ One example is CMV, which precisely adapts to the host by co‐evolving with the immune system and establishing a state of detente.^73^ The latent virus sporadically reactivate when the host immune system is compromised, resulting in the generation of highly functional and resilient CD8^+^ T cells, as well as memory inflation.^65^, ^74^ On the other hand, partial adaptation to the host leads to persistent viral replication and antigen expression, causing cellular damage, immune dysfunction or exhaustion.^75^, ^76^ Consequently, severe chronic or progressive diseases could be ensued, such as comorbidity of human immunodeficiency virus and hepatitis B virus.^77^, ^78^, ^79^ To gain deeper insights into the alternative immune responses triggered by latent and persistent viral infections, researchers have established a commonly utilized murine model with LCMV infection, which brings exhausted CD8^+^ T (T_EX_) cells.^24^, ^80^ It is well established that exhaustion occurs due to sustained stimulations by cognate antigens in driving CD8^+^ T cells to become reliant on antigenic signals for their survival.^81^ Functionally, CD8^+^ T_EX_ cells exhibit reduced proliferative capacity, cytotoxicity, and cytokine secretion.^82^ In both murine models and human patients, CD8^+^ T_EX_ cells are mainly characterized by elevated expression of the inhibitory receptors, include programmed death‐1 (PD‐1), CTLA‐4 and LAG3.^83^, ^84^ Besides, by lacking of KLRG1, CD127 and CD45RO (CD44 for murine model), the differentiation, secretion and proliferation abilities of CD8^+^ T_EX_ cells are limited.^85^ More specifically, a wide variety of other inhibitory markers were detected in CD8^+^ T_EX_ cells, such as CD62L, TIGIT, TIM3 and CD160, thus prohibiting priming and activation.^86^ Meanwhile, the effective function of CD8^+^ T_EX_ cells gradually declines, accompanied by the losses of IL‐2, TNF, and IFNγ productions due to the irregular expressions of T‐bet and EOMES (Table 1).^87^, ^88^ Except the way of development of viral infection, exhaustion could be considered as the opposite status of memory inflation.

## 3 POTENTIAL MECHANISMS OF MEMORY INFLATION

In some infected individuals, the proportion of inflated CD8^+^ T cells can reach up to over 50% of total CD8^+^ T cells and last for years.^89^ In murine models, the ratio of inflated CD8^+^ T cells attains over 70% at 1 month after CMV infection, while it comes to around 50% over 3 months post‐infection and stabilizes with this level for years.^40^ However, on account of the dynamics of host immune landscape, inflated CD8^+^ T cells have been monitored to differentiate with numerous unforeseen consequences.^90^ And up to date, the exactly mechanism that responsible for inflation of CD8^+^ T cells has not been clearly elucidated.^91^ Here, we go through current knowledge for the potential aetiologies of memory inflation under viral infection.

### 3.1 Intrinsic and extrinsic roles of inflated CD8 ^+^ T cell formation

It is well‐recognized that the formation of inflationary CD8^+^ T cells has been ascribed to both intrinsic and extrinsic contexts, mainly depend on the expressions of proteasomes in immune cells and properties of viral epitopes, which affect the binding avidity and TCR affinity.^92^ Current research studies identified that multi‐epitopes from viruses have the ability to provoke inflationary responses via inducing constitutively expression of proteasomes in non‐haematopoietic cells of host.^93^ Studies on downstream proteins of proteasomes indicated that M27 favours peptide processing for MHC I presentation, which may enhance the activation of T cells to further form memory inflation via inhibiting the induction of immunoproteasomes.^94^ On the other hand, M27 derived from CMV could also lead to ubiquitination of viral proteins and provide stress responses in attenuating viral activities and increasing cell viabilities in against infection.^95^ In spite of CMV, this ubiquitin‐proteasome pathway was also observed to participate in the formation of memory T cells during the degradation of core proteins to eliminate replications of hepatitis B and influenza A virus.^96^, ^97^, ^98^ Interestingly, the COVID‐19 virus were found to follow a similar manner to trigger memory inflation of T cells in certain conditions.^99^ Actually, epidemiological reports from the past pandemic of COVID‐19 revealed that considerable numbers of individuals were observed to possess numerous virus‐specific CD8^+^ T_E/M_ cells in circulating system for almost 1 year after infection, although most of them were evaluated to be asymptomatic patients.^100^ Therefore, regardless of clinical symptoms, inflationary responses can be induced by viral peptides generated from the constitutive activation of proteasomes during the process of immune protection.^93^ And memory inflation is believed to be a significant phenomenon that associated with the expression of proteasomes in non‐haematopoietic cells.^101^ In addition, the CD8^+^ T cell subsets to recognize m139_419–426_ were monitored to have a phase of contraction with memory‐like phenotypes after rapid expansion, whereas no contraction phase is observed in the expansion of CD8^+^ T cells to recognize epitopes that located in M38_316_ and immediate early proteins (IE).^45^ All of those epitopes were detected to be eliminated gradually due to the clearance of virus.^102^ Therefore, we assumed that the changes of CD8^+^ T cell metabolism and function might perform as additional intrinsic manners to bias memory inflation.

In spite of the proteasome networks and metabolic changes in host's cells, viral epitopes have been identified to elicit persistent responses in priming T cells and then shape robust characteristics of memory inflationary phenotype.^103^, ^104^ For example, CMV lower matrix 65‐kDa phosphoprotein (pp65) (NLVPMVATV) epitope, which is usually considered as the marker for acute infection and presented by HLA‐A*0201 (A2/NLV), could initiate primed CD8^+^ T cells differentiated into effector and MP subsets, thus further give rise to the expansion of CD8^+^ T_E/M_ cells.^105^ Moreover, studies on human CMV (HCMV)‐encoded open reading frames‐mediated recall responses revealed that pp65 and UL123 (IE) could be recognized by more than half of the infected cohort and provoke greater responses in comparing to other antigens, such as pp150, gB, gH, pp50, and pp28.^104^ Thus, pp65 and IE are not only responsible for clearance of virus‐infected cells at early stage, but also associated with the immune‐dominant antigens for memory CD8^+^ T cells generation.^104^ Meanwhile, recent studies have demonstrated the decisive role of pp71 in enhancing the IE1‐derived peptides presenting by HLA class I molecules, thereby facilitate the formation of inflated CD8^+^ T cells.^106^ By contrast, IE2‐pp86 was reported to downregulate chemokine expression during acute phase, and then impede the recruitment of CD8^+^ T cells, as well as the strength of anti‐viral immune response.^107^ Similarly, gpUS18 and gpUS20 were also observed to facilitate the establishment of chronic infection, but not memory inflation by affecting NK cell function in acute phase (Table 2).^108^ In chronic phase, gpUS2, gpUS3, and gpUS11 jointly blocked the HLA class I pathway to inhibit proliferation and activation of memory CD8^+^ T cells.^109^, ^110^ Although numerous viral antigens were confirmed to participate the differentiation of CD8^+^ T cells with memory inflated phenotype, distinguish features of those antigens in serving CD8^+^ T cells' trajectories remain unveiled.^111^ One hypothesis of peptide selection for memory inflation is the discrepancies in epitope‐display between priming and further differentiations, as well as expansions.^112^, ^113^ In acute phase of MCMV‐infection, C57BL/6 murine CD8^+^ T cells' responses were elicited to against at least 24 epitopes derived from 18 proteins, like M45, m141, M86, M57, M78, and M86.^28^, ^45^ Nevertheless, except promotion of host defences against infection, which facilitates long‐term antigen exposure and T cell recognition, none of those epitopes is directly associated with memory inflation.^114^, ^115^ On the other side, according to TCR specificities of inflated CD8^+^ T cells after infection, five singular epitopes (m139 419–426, M38 316–323, IE3 416–423, IE3 461–475, and M102 486–500) were found to dominate clonal expansion and recall response (Table 2).^28^, ^45^ Notably, two epitopes also encoded by IE3 (IE3_416–423_ and IE3_461–475_) are readily detectable in acute infection but exhibit constitutively expression at chronic phase.^45^ This sequential expression pattern mainly depends on the virus duplication and spreading, but whether they determine the strength and/or persistence of memory inflation is not fully known.^116^

**TABLE 2:** Antigen epitopes from CMV and their potential intrinsic roles.

Antigen epitopes	Roles and mechanism
*HCMV*	
IE1‐pp71	Enhancing the presentation and supporting CD8+ recognition
IE2‐pp86	Inhibits the expression of chemokine
pp65	Inflationary phenotype; Inhibits antiviral gene expression and IFN signalling
gpUS3	Downregulation of HLA class I pathway
gpUS2 and gpUS11	Degrade HLA class I molecules
gpUS18 and gpUS20	NK cell function
*MCMV*	
M45	Acute phase of CD8+ T cells responses
m139 and M38	Inflationary phenotype

Except of the antigen specificities, initial viral titrations were revealed to exert promising effects for inflationary CD8^+^ T cells generation.^46^ Low‐dose (less than 1 × 10^1^ PFU) inoculation with CMV could dampen the inflated CD8^+^ T cells accumulation and lead to expansion of T_CM_ phenotype for secondary response, which is more likely to happen in latency‐dominant response during chronic infection or vaccination.^117^, ^118^ Meanwhile, at the period of post‐infection, few CMV latency is found to remain in non‐haematopoietic cells, including vascular endothelial, lymph node interstitial, lung, and bone marrow cells, which are confirmed to be essential for driving antigen‐specific T_CM_ and resident memory CD8^+^ T (T_RM_) cells proliferation, thus reducing T_E/M_ population in circulating system.^43^, ^117^, ^119^ By contrast, results from murine models indicated that CMV infection with a high‐dosage (more than 1 × 10^4^ PFU) not only provokes a rigorous anti‐viral response, but also sharply deflates circulating CD44^low^CD62L^+^KLRG1^−^CD8^+^ T_N_ cells and increases accumulation of CD8^+^ T_E/M_ cells with CD44^high^CD62L^−^KLRG1^+^ phenotype.^89^, ^117^ Presumably, NK cell‐mediated cytokine storm is supposed to benefit the differentiation of CD8^+^ T_E/M_ cells via antigen‐independent manner.^120^ Moreover, in the absence of immune‐evasion protein polymers, enhanced cross‐presentation might play a crucial role in priming CD8^+^ T_N_ cells to T_E/M_ phenotype thus facilitate memory inflation under excessive virus‐induced inflammation.^121^ This notion is supported by the evidences from BATF3‐deficient mice with viral infection, by lacking of dendritic cells (DCs), disabled cross‐presentation affects a wide range of epitopes‐dependent differentiation of CD8^+^ T_E/M_ cells.^122^, ^123^ Simultaneously, studies by using bone marrow chimeric mice with MHC or TAP knock‐down in DCs revealed that memory inflation is severely blocked after the impairment of cross‐presentation, but priming remains intact.^124^

Overall, many sophisticated triggers have been identified to contribute for the development of memory inflation after CMV infection. In spite of the importance of antigen persistence in the formation of memory inflation, studies also revealed that sporadically reactivated virus from latency could be processed by the constitutive proteasome and presented by MHC class I molecules.^74^ In addition, both infected somatic cells and cross‐primed/cross‐dressed DCs are observed to present viral antigens via endogenous antigen presentation pathway.^110^, ^124^ Besides, TCR signalling and co‐stimulatory molecules are contributing to inflationary CD8^+^ T cell differentiation in concomitant with multiple inflammatory cytokines, including IFNγ, IL‐12, and IL‐7.^125^ It is worth to mention that activation of CD4^+^ T cells are considered to be one of the decisive factors in the formation of inflationary CD8^+^ T cell population as well.^64^, ^126^ Collectively, in view of the multi‐dimensional intrinsic factors and external conditions to orchestrate memory inflation, further investigation should be conducted to emerge the under mechanism for this apparent increase in heterogeneity of CD8^+^ T cells.

### 3.2 Functional proteins to maintain the inflationary populations

As the transcription factor for controlling proliferative potential, self‐renewal capacity, as well as terminally differentiated T cells generation, Tcf1 is considered to be one of the most important proteins to maintain memory inflation.^127^ For keeping pluripotency of CD8^+^ T_N_ and primed T cells at the initiation of memory inflation after infection, Tcf1 constitutively expresses in T cells with high proliferative activities.^128^ Along with T cell differentiation, Tcf1 is detected to eliminate in terminal differentiated cells, such as T_E_ and T_EMRA_ cells, which show a relatively short life‐span and last for a few days in both circulating system and tissues.^127^, ^129^ Therefore, Tcf1 is recognized as additional marker for characterizing inflated CD8^+^ T cell subsets in associated with CD127 expression and the absence of KLRG1.^130^ Meanwhile, this less‐differentiated population is mostly composed by antigen‐specific CD8^+^ T_E/M_ cells that possesses self‐renewal ability through an antigen‐independent manner, and plays vital roles in the maintenance of inflationary population.^41^, ^131^ Besides, recent studies demonstrated that Tcf1 could also trigger CD8^+^ T_CM_ cells in differentiating to inflationary T_E/M_ cells during sporadic reactivation after virus clearance.^11^, ^132^ Furthermore, inflationary Tcf1^+^CD8^+^ T cells from lymph nodes asymmetrically differentiate into Tcf1^−^ effector‐like T cells, which is necessary to sustain memory inflationary population.^42^ Actually, on basis of epigenetic inheritance, Tcf1 expression is varying depending on the dynamic changes of cell status, and there is still controversial that Tcf1 signalling might have alternative functions in contributing the progression of memory inflation.^133^

CD127 is a well‐documented subunit of IL‐7 receptor that controls T cell differentiation in secondary lymphoid organs.^134^, ^135^ Accordingly, the expression of CD127 mainly determines the phenotypic differentiation of T cells after antigen stimulation.^42^ By receiving IL‐7 from fibroblastic reticular cells, CD127^+^CD8^+^ T cells further differentiated into CD8^+^ T_MP_ cells, which are responsible for the origin and expansion of inflationary T cells.^136^ Moreover, during the acute phase of viral infection, IL7/CD127 signalling additionally enhanced the proliferative and differentiative abilities of CD8^+^ T_MP_ cells, thus partially resembled CD62L^+^CD27^+^ T_CM_ cells with functions that generate inflationary CD8^+^ T cells in triggering secondary response.^21^ Consequently, apart from the surface marker for CD8^+^ T_E/M_ cells, the expression of CD127 retains the homeostasis of inflationary CD8^+^ T cell expansion.^137^

KLRG1 is originally identified as the inhibitor for Cadherin family.^138^ And KLRG1 expression in antigen‐stimulated T cells implies the failure of priming.^139^ On the contrary, the absence of KLRG1 could unchain the secreting of inflammatory cytokines in CD8^+^ T cells, such as IFNγ and TNF.^140^ Studies on establishment of memory inflation with CMV infection confirmed that early‐primed KLRG1^−^CD8^+^ T cells directly contribute to form the trajectory from high avidity T_CM_ precursors to inflationary T cell pool at latency phase.^89^ The above observations indicate that KLRG1 negatively regulates inflationary functionality, and acts as a checkpoint in preventing excessive secondary immune responses.^141^

Despite of the pivotal functions to induce M2 macrophage polarization and mediating phagocytosis, high level of CX3CR1 also brings both cytotoxicity enhancement and pro‐inflammatory cytokine secreting in CD8^+^ T_E/M_ cells.^142^ Unlike CD44, which is mainly detected in murine effector‐like T cells, CX3CR1 is often combined with CD27 expression to determine memory T cells in both mice and human at the early memory stage after CMV infection.^143^ Meanwhile, CX3CR1 signal is demonstrated to participate in CD8^+^ T cell differentiation during viral infection, including expansion of CD8^+^ T_E/M_ cells from T_E_ and T_CM_ cells, as well as stimulation of T cells with naïve status towards a more differentiated state.^143^ Sustained CX3CR1 expression in T_CM_ and T_E/M_ populations gives rise to enhanced virus clearance at secondary infection.^144^ Besides, diminished CD27 expression on inflationary CX3CR1^+^CD8^+^ T cells occurs at the time of T_EMRA_ population development.^145^ Pathologically, CD8^+^ T_E/M_ cells would quickly expand and differentiate into large numbers of CD8^+^ T_EMRA_ cells in responding to secondary infection, and CD27 likely to play a role in tapping asymmetric differentiation of T_E/M_ cells for memory inflationary maintenance and producing CD8^+^ T_EMRA_ cells via CD70 stimulation.^146^ And the conversion of CD27^dim^ from CD27^+^ is considered as a sign of losing long‐term memory in inflated CD8^+^ T cells.^147^

## 4 MEMORY INFLATION AND DISEASES

### 4.1 Autoimmune neuropathy

Guillain‐Barré syndrome (GBS) is a rare autoimmune polyneuropathy that is characterized by autoantigen specific immune responses and T cells accumulation in peripheral nervous system.^148^ Clinically, ascending demyelination of peripheral nerve tissues would cause flaccid paralysis, neuropathic pain, as well as respiratory repression with an approximately 3–7% of the mortality rate.^149^ In addition to genetic susceptibility, environmental factors have been recognized as significant contributors to the development of GBS, with over 65% of GBS cases were found to occur after infectious diseases or vaccination.^150^, ^151^ Upon repeated stimulation and/or infection, dynamic changes of host immune background were believed to exacerbate potential cross‐reactivity between pathogens and antigens of nervous system in eliciting autoimmunity.^152^, ^153^ This autoimmune responses usually target proteins that specifically expressed in myelin and/or axons of the peripheral nervous system, resulting in demyelinating or axonal damage.^154^

Other than *Campylobacter jejuni* (24.6%), many viruses are well‐documented to associate with GBS onset, including CMV (12.4%), Epstein–Barr virus (1.3%) and Zika virus.^155^ Theoretically, virus‐induced highly proliferative CD8^+^ T cells are recruited at the injury site from circulating system and facilitate the progression of autoimmune peripheral neuropathy (APN).^150^ Latest observations have revealed that these antigen‐specific CD8^+^ T cells exhibit cytolytic phenotypes and directly participate both demyelination and axons damage.^156^ By utilizing a transgenic mouse model, Yang et al. revealed that constitutively expression of *b7*.*2* with *cd4* knockout results a spontaneous APN, which mimics most clinical and pathological features of GBS, and CD8^+^ T_E/M_ cells dominant the pathogenesis of disease at the lesion site.^157^ In parallel, partial sciatic nerve ligation boosted M1 macrophage polarization could also accelerate the onset of APN via provoking CD8^+^ T_E/M_ cells in *cd4* knockout mice.^150^ However, apart from the phenotypic identification of CD8^+^ T_E/M_ cells in triggering GBS, further studies should be done on the similarities of TCR repertoires between CD8^+^ T_E/M_ cells in recognizing virus and autoantigen.^158^, ^159^ In addition, due to the delayed memory inflation after infection, there is a difficulty on occasion to determine the association between viral infection and APN, but investigations have demonstrated the 8 years earlier on average onset of GBS in population with CMV infected history by comparing with uninfected patients.^160^ Collectively, inflated CD8^+^ T cells with effector/memory phenotypes from viral infection be responsible for GBS onset and progression.

There is a long‐time debate that whether CD8^+^ T cells act as a prominent factor in inducing multiple sclerosis (MS), which is considered as an incurable autoimmune disease in the central nervous system (CNS).^161^, ^162^ In recent years, clonal expansions of CD8^+^ T cells have been found to dominate the infiltrated T cell population in active MS lesions of patients.^163^ Meanwhile, the abundance of CD8^+^ T cells surpasses that of CD4^+^ T cells at all stages of disease.^164^ And knockout of CD4^+^ T cells in murine models of MS not only fails to alleviate the disease progression, but also actually exacerbates the symptoms.^165^ Moreover, in experimental autoimmune encephalomyelitis murine model, the most widely used murine model to mimic MS, restimulated CD8^+^ T cells from MCMV co‐infection group exhibit higher responsive rate in comparing with CD8^+^ T cells from the group that solely induced by MOG35‐55.^166^ Additionally, upon restimulation, CD8^+^ T cells showed less specific to MCMV antigenic peptides, but exhibited characteristics that similar with T_E/M_ and T_EMRA_ populations in responding to autoantigens from CNS.^166^ Evidence from MS patients also revealed that expanded CD8^+^ T cells with Granzyme B secreting could recognize epitopes from both CMV and autoantigens to promote disease relapse and treatment‐resistance.^167^ Phenotypically, this clonal‐expanded CD8^+^ T cells from patients are terminally differentiated and resemble the features of T_EMRA_ as well.^18^

**FIGURE 2:**
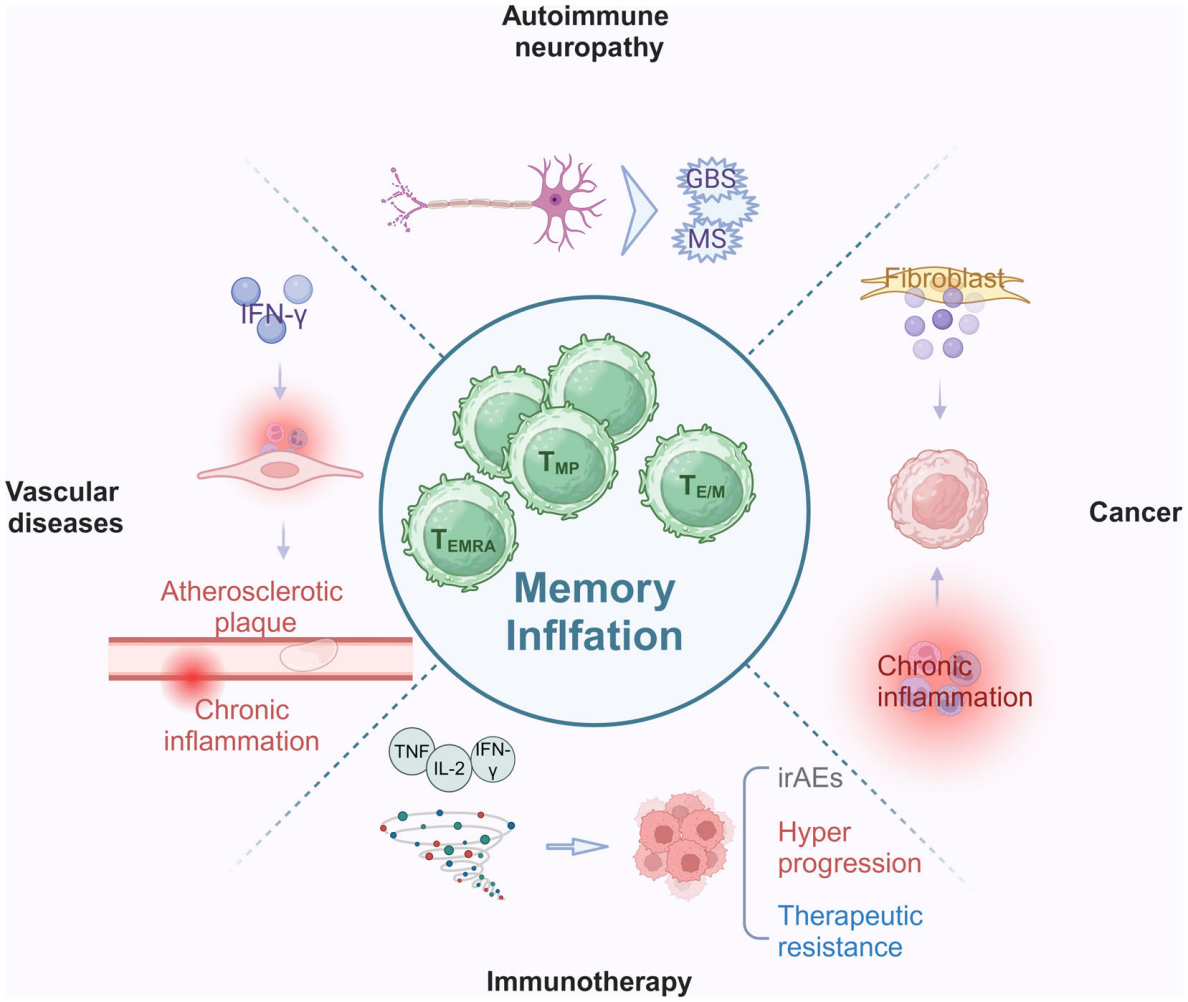
Memory inflation and diseases. Increased CD8^+^ T_E/M_ and T_EMRA_ cells when memory inflation occurs are strongly associated with autoimmune neuropathy, cancer, and vascular diseases, also they can affect the effectiveness of immunotherapy. GBS, Guillain‐Barré syndrome; MS, multiple sclerosis; irAEs, immune‐related adverse events. T_MP_, Memory precursor T cells; T_E/M_, Effector/Memory T cells; T_EMRA_, terminally differentiated effector memory T cells.

In summary, mounts of studies indicated that the development of autoimmunity in nerve system is not only influenced by genetic background, but could also be provoked by environmental factors, such as viral infection.^168^, ^169^ Although limitation on directly obtaining the evidence from lesion sites of nervous system, but the observations of peripheral CD8^+^ T_E/M_ and T_EMRA_ cells in mediating autoimmunity at both patients and murine models implied that memory inflation acts a key role of APN development.^150^, ^158^ Accordingly, the dynamic change of peripheral CD8^+^ T cell phenotypes after CMV infection are presumably responsible for the pathogenesis and progression of certain autoimmune neuropathies.^170^ Up to date, cross‐reactivity from virus‐specific inflated CD8^+^ T cells via molecular mimicry is believed to be the leading hypothesis of sequestered antigen recognition and autoimmune derivation.^29^, ^171^ Due to the significant roles of inflated CD8^+^ T cells in GBS/AIDP and MS onsets, it would be necessary to further dissect the potential regulatory roles of T_MP_, T_E/M_, and T_EMRA_ populations in disease prevention and treatment (Figure 2).

### 4.2 Cancer and immunotherapy

Epidemiologically, over 13% of cancer cases were attributed to infection‐related factors, which are accounting for 64% of them with viral infections.^172^, ^173^ Despiting the gene integration‐caused mutation, a high prevalence of CMV post‐infection was found in patients with various types of cancer.^174^ And numerous studies have provided evidence that CMV may facilitate tumour progression and contribute to poor prognosis by remodelling tumour microenvironment, impacting T cell phenotypes, and suppressing the effects of antitumor therapy.^175^, ^176^, ^177^ In patients with multi‐forms of glioblastoma (GBM), CMV antigen expression and its role in tumorigenesis have emerged as promising targets for immunotherapy (Table 3). During the acute phase of CMV infection, fibroblasts exhibit increased secretion of pro‐inflammatory factors, including IL‐6, TNFα, CCL2, CCL5, and GM‐CSF. Conversely, the secretion of anti‐inflammatory cytokines such as IL‐4, IL‐28A, CCL24 and SCF is reduced.^178^ Both IL‐28A and SCF were confirmed to possess anti‐tumour effects, while higher levels of CCL24 could facilitate the prognosis in breast cancer.^179^, ^180^ Meanwhile, prolonged and repeatedly infection by CMV leads to progressive depletion of CD8^+^ T_N_ cells and increase of CD8^+^ T_E/M_ and CD8^+^ T_EMRA_ populations with viral antigen specific profiles, which might potentially devastate the anti‐tumour responses from T cells.^181^, ^182^ After virus clearance, long‐term memory inflation was also demonstrated to contribute the formation of chronic inflammation thus improve the susceptibility of cancer.^183^, ^184^ And in cancer patients, accumulation of non‐tumour antigen specific CD8^+^ T cells with inflated phenotypes in peritumoral lesions impairs anti‐tumour responses and induces the progression of cancer.^185^ Evidences from clinic examinations revealed that excessive inflated CD8^+^ T cells in peripheral system suggest a prominent severe symptoms and poor prognosis of patients.^186^, ^187^ The presence of cloned proliferating immune cells in GBM patients compromises the effectiveness of the immune system in mounting an anti‐tumour response.^188^ For refractory and recurrent cases, a promising approach is observed in combination of antiviral therapy with chemotherapy.^189^, ^190^ More importantly, as the sign of memory inflation, reduction of CD8^+^ T_N_ cells could dampen the immune responses for tumour antigens and neoantigens, thus further give rise to poor prognosis of cancer patients.^191^ Conversely, by recruiting 28 patients with primary GBM for adjuvant therapy using autologous CMV‐specific T cells expanded in vitro, CMV‐specific T cells regained immune function and had the potential to trigger a bystander effect in responding to tumour‐associated antigens.^192^ This particular phenomenon maybe derived from the cross‐presentation that is due to the isolation of immune milieu in CNS.^193^ Moreover, it appears that peripheral inflationary CD8^+^ T cells that specific for CMV are less exhaustion and functionally energetic in comparing with tumour‐antigen specific T cells from draining lymph nodes of patients with cancer.^194^ Accordingly, high frequency of inflationary T cell population from CMV infection may not simply be a “destroyer” or “bystander” in patients with cancer, further investigations are needed to dissect the attribution of viral‐mediated inflated CD8^+^ T cells in cancer progression.

**TABLE 3:** Current clinical trials using cytomegalovirus‐targeting immunotherapy for patients with glioblastoma (accessed on 23 May 2021, ClinicalTrials.gov).

Intervention	Enrolment	Phase	Duration	NCT number	Status
Safety and tolerability of VBI‐1901 vaccine candidate in patients with recurrent GBM	38	I/II	2017–2021	NCT03382977	Active, not recruiting
Autologous cytomegalovirus‐specific cytotoxic T cells combined with temozolomide (TMZ)	65	I/II	2016–2021	NCT02661282	Active, not recruiting
TMZ^+^ cytomegalovirus peptide (PEP‐CMV) vaccine	70	I	2016–2021	NCT02864368	Active, not recruiting
Cytomegalovirus pp65‐LAMP mRNA‐loaded dendritic cells (DCs) with or without autologous lymphocyte transfer, tetanus toxoid	42	I	2006–2022	NCT00639639	Active, not recruiting
pp65‐shLAMP DC with GM‐CSF	120	II	2016–2024	NCT02465268	Recruiting
TMZ^+^ human CMV pp65‐LAMP mRNA‐pulsed autologous DCs	100	II	2015–2020	NCT02366728	Active, not recruiting
TMZ^+^ Human CMV pp65‐LAMP mRNA‐pulsed autologous DCs containing GM‐CSF^+^ Tetanus‐Diphtheria Toxoid (Td)^+^ GM‐CSF^+^ 111‐Indium‐labeling of Cells for in vivo Trafficking Studies	48	II	2019–2023	NCT03927222	Recruiting
Temozolomide^+^ Radiotherapy with or without Valganciclovir	220	II	2019–2024	NCT04116411	Recruiting

Abbreviations: GM‐CSF, granulocyte–macrophage colony‐stimulating factor; HER, human epidermal growth factor receptor; HER‐CAR, HER chimeric receptor; LAMP, lysosome‐associated membrane protein.

Cancer immunotherapy utilizes the inherent capabilities of the immune system to activate or enhance immune responses against cancer cells.^195^ Common immunotherapy approaches include immune checkpoint inhibitors (ICIs), chimeric antigen receptor T‐cell therapy, and vaccine therapy.^196^, ^197^, ^198^, ^199^ And ICIs is the most widely adopted immunotherapeutic method that achieves remarkable success in certain types of cancer, including melanoma,^199^ non‐small cell lung cancer,^200^ and haematological malignancies.^201^ Nevertheless, on basis of the new indications in continually expanding, the gross response rate of ICIs is below 30%, which represents over 70% of the patients have to experience therapeutic resistance, immune‐related adverse events (irAEs), as well as hyper progression.^202^, ^203^ Recent studies from clinics indicated that the same ICIs therapeutic strategies exhibit alternative effects in patients with similar tumour burden and immune checkpoint expression level.^204^ As both cytolytic cells for tumour and target cells of ICIs, CD8^+^ T cell is believed to play decisive role in affecting the anti‐tumour effects of ICIs.^205^

It is well‐documented that accumulation of tumour antigen non‐specific CD8^+^ T_E/M_ and T_EMRA_ cells and decreasing of CD8^+^ T_N_ and tumour antigen specific T_MP_ cell populations lead to diminish the tumour cytotoxicity.^206^, ^207^, ^208^ As the function of ICIs is mainly unchaining the inhibitory effect of immune checkpoint from co‐stimulatory signalling pathway, high levels of non‐specific CD8^+^ T_E/M_ and T_EMRA_ population could bring excessive systemic IL‐2, TNF, and IFNγ secreting, thus promote cytokine storm.^209^ Additionally, less CD8^+^ T_N_ cells imply a relatively weaker clonal expansion of T cells for tumour specific immune responses.^182^ As a result, this particular immune background might increase the risk of therapeutic resistance and irAEs of ICIs.^210^ Other than epigenetic alternations, CMV‐mediated memory inflation is considered as the most likely trigger to reduce CD8^+^ T_N_ and T_MP_ cell populations, as well as generate large amounts of CD8^+^ T_E/M_ and T_EMRA_ cells with virus‐antigen specific TCR.^211^, ^212^ Hence, in considering the highly infectious rate of CMV worldwide, memory inflation of CD8^+^ T cells is supposed to be one of the primary cause in inducing therapeutic resistance of ICIs treatment.^213^ Likewise, due to the increased numbers and enhanced activity of inflated CD8^+^ T cells, CMV infection has also been repeatedly reported to be responsible for recurrent and refractory irAEs in patients after receiving ICIs treatment.^214^, ^215^, ^216^ Unfortunately, although it is clear that post‐CMV infection impairs the efficacy of ICIs, but the exactly CD8^+^ T cell phenotypes in association with their immune functions to be attributed in responsible for certain poor prognoses of patients after ICIs treatment are still enigma (Figure 2).

Meanwhile, data from both in vitro and in vivo studies showed a promising result that CMV‐specific CD8^+^ T cells could significantly eliminate cancer cells.^217^, ^218^ This finding might provide an alternative immunotherapy approach for cancers. On basis of the self‐renewal ability, CMV‐specific CD8^+^ T cells possess cytolytic activities without further stimulation or manipulation.^217^ ex vivo investigation confirmed that memory inflated CMV‐specific CD8^+^ T cells from peripheral blood could directly secret pro‐inflammatory cytokines.^219^ Although these subsets exhibit less specificity for anti‐tumour responses, but their secreting features might partially release tumours from immunosuppressive microenvironment in certain circumstances.^219^ Furthermore, several tumour types are found to contain CMV peptide epitopes on account of the virus latency, which could engage peripheral CD8^+^ T cells to expand into memory inflated phenotypes, and further improve the efficacy of CD8^+^ T cell‐based anti‐tumour responses, as well as ICIs treatment.^220^ Hence, we could not entirely exclude the possibility that CMV infection‐mediated memory inflation of CD8^+^ T cells also facilitate to tumour microenvironment with non‐specific manner.

### 4.3 Vascular diseases

Cardio/cerebrovascular diseases are among the leading causes of mortality in the modern era.^221^ Despite extensive histopathological research studies, arterial atherosclerosis has emerged as well‐documented occasionally pathological changes in both heart and brain strokes.^222^, ^223^, ^224^ Mechanistically, most arterial atherosclerosis is observed to display a chronic characteristic in affecting the bloodstream at large and medium‐sized arteries.^225^, ^226^ The formation of thromboses in severely stenosed arteries eventually results in blood flow restriction and tissue hypoxia.^227^ Consequently, irreversible damage occurs in tissues and organs under insufficient blood supplies.^228^, ^229^ This process was also recognized as the cause of myocardial infarction, heart failure, cerebral infarction, and peripheral vascular diseases.^230^, ^231^ Numerous prospective and retrospective studies revealed a significantly higher risk of atherosclerosis associated with diabetes, hyperlipidaemia, hypertension, smoking, insulin resistance, and family history.^232^ However, the initial aetiology of atherosclerosis, the underlying cellular and molecular driving factors, and the influence of the external environment on its progression remain unclear.

Other than unregulated retention of lipoproteins‐mediated cholesterol buildup, emerging evidences from both basic and clinical research studies suggest that atherosclerosis should be redefined as a chronic inflammatory disease.^233^ A constitutive sub‐inflammation is considered as a certain congenital factor to recruit immune cells in infiltrating into the atherosclerotic plaque.^234^ Interestingly, epitopes from antigens of atherosclerosis, such as apolipoprotein B (ApoB) and LDL have similar immunological features with viral antigens, including CMV, HCV, and HPV.^216^ By recognizing the core proteins of ApoB and LDL, immune cells led to atherosclerosis that similar with an autoimmune manner.^235^ Meanwhile, both repeated and post viral infection are supposed to have close relationship with atherosclerosis onset.^236^, ^237^, ^238^ Consequently, recent studies have highlighted the crucial role of CD8^+^ T cell heterogeneity in the development of atherosclerosis.^226^, ^230^, ^233^, ^239^ Observations from patients with coronary heart disease revealed that both peripheral and atherosclerotic plaques had increased cytotoxic T cells, which possess similar features of inflated CD8^+^ T cells and further accelerated disease progression.^239^ In advanced atherosclerosis, other than cells belong to innate immune system, excessive expansion of infiltrating CD8^+^ T cells was found to dominate the immune microenvironment in lesion areas.^240^ Recent studies have drawn a possibility explanation of CD8^+^ T cells promote lesion progression and plaque instability.^241^ By secreting cytokines, CD8^+^ T cells with effector memory and/or terminal differentiated effector memory phenotypes exacerbate the inflammation and cause cytotoxicity to endothelial and other somatic cells.^241^, ^242^ Thus, significant attention has been directed towards the involvement of inflated CD8^+^ T cell subsets in atherosclerosis.^243^ Although activated CD8^+^ T cells are demonstrated to participate in atherosclerosis progression, but up to date, no evidence is obtained to show the direct effect of CD8^+^ T cells in facilitating dieses onset.^239^

Other than inducing of viperin in interacting with platelets, CMV is the major factor to generate memory inflationary immune background.^244^, ^245^, ^246^, ^247^ And numerous clinical studies have demonstrated that dynamic change of CD8^+^ T cell phenotypes is one of the decisive factors for vascular diseases after CMV infection.^248^, ^249^ Due to the expansion of inflationary CD8^+^ T cells with IFNγ secreting, a non‐selective inflammation might promotes atherosclerotic plaque formation.^250^ According to the antigen‐independent manner of self‐renewal feature and enhanced infiltrated ability, CD8^+^ T_E/M_ cells from memory inflation would prompt sustained chronic inflammation in the lesion sites of atherosclerosis.^251^ Moreover, eliminating of CD8^+^ T_N_ cell populations in memory inflation could also be a risk factor for cardio/cerebrovascular diseases, as CD8^+^ T_N_ cells contributing to alleviate disease progression.^81^ By adoptive transferring ApoB‐related peptide (p210) immunized CD8^+^ T_N_ cells into ApoE^−/−^ mice, strengthening innate immune responses were monitored in sites of atherosclerotic plaques.^81^ Similarly, primed CD8^+^ T cells possess higher lytic activity to inhibit macrophage and pathological CD4^+^ T cells in ApoE^−/−^ mice when vaccinated with the p210 vaccine.^239^, ^252^ However, limited research studies have explored the specific immune responses caused by CMV infection and their impacts on atherosclerosis. Simultaneously, the understanding of alternative roles of CD8^+^ T cell subsets in atherosclerosis progression is also unrevealed (Figure 2).

## 5 CONCLUDING REMARKS

Current studies have shown that CMV‐induced memory inflation involves both innate and adaptive immune responses in leading an antigen‐independent differentiations and immune function changes of CD8^+^ T cells.^129^ The expansion and stabilization of circulating CD8^+^ T_E/M_ cell populations could be considered as the major sign of memory inflation.^123^ Consequently, these antigen‐specific CD8^+^ T cells with memory inflation phenotype play a crucial role in both antigen‐specific and non‐selective immunity.^111^ And the onset and progression of various diseases, including autoimmune disorders, cancer, and cardio/cerebrovascular disorders, as well as resistance of immunotherapy have been demonstrated to link with inflationary CD8^+^ T cells.^150^, ^188^, ^208^, ^250^ However, the underlying mechanisms for maintaining memory inflation, and the cellular characteristics and key molecules of memory inflation in resulting disease development, remain largely unexplored.^59^, ^89^, ^112^, ^253^ Further investigations are required to clarify these discrepancies in order to develop novel therapeutic strategies for correcting this harmful change in the host immune background. Meanwhile, due to the inconsistencies in the phenotypic and functional features of memory‐inflated cells between human patients and rodent models, the potential mechanisms that are responsible for memory inflation initiation, such as intrinsic and extrinsic properties of viral antigens, still need further exploration.^40^, ^254^, ^255^ Immune‐modulating approaches and new animal models will be provided by further investigating to prevent the development of memory inflation.

Overall, this review highlights the definition and origination of memory inflation, the distinctive phenotypes and functions of memory‐inflated cells. Owing to the significance of CMV‐mediated host immune background heterogeneity, and its potential impacts on autoimmune disorders, cancer, cardio/cerebrovascular disorders, and resistance of immunotherapy, the better understanding of memory inflation provides us an opportunity to beyond the acute phase of viral infection, and sheds a light on the long‐term influences of post‐infection in host immune system.

## AUTHOR CONTRIBUTIONS

Mu Yang conceived the review article. Yanfei Li and Jie Xiao contributed to the initial drafting of the manuscript, table preparation, visualization, and overall editing. Mu Yang and Chen Li helped to modify the manuscript. Mu Yang and Chen Li contributed to conceptualisation, funding, overall supervision, and supported review development, overall editing, and critical overall manuscript revision. All authors read and approved the final manuscript.

## FUNDING INFORMATION

This study was supported by grant ZYGX2021YGCX005 (to M.Y.) from the Medicine and Engineering Cross‐Innovation on Oncology of University of Electronic Science and Technology of China, grant 82300276 (to C.L.) from the National Natural Science Foundation of China, grant TB2022085 (to C.L.) from the Sichuan Postdoctoral Foundation, grant 2023NSFSC1643 (to C.L.) and grant 2023ZYD0045 (to M.Y.) from the Sichuan Natural Science Foundation, and Sichuan Young Talents Foundation (to M.Y.).

## CONFLICT OF INTEREST STATEMENT

The authors declare no potential conflict of interest.
