# Correction to “Memory Inflation: Beyond the Acute Phase of Viral Infection”

**DOI:** 10.1111/cpr.70054

**Published:** 2025-05-05

Y. Li, J. Xiao, C. Li, and M. Yang, “Memory Inflation: Beyond the Acute Phase of Viral Infection,” *Cell Proliferation* 57, no. 12 (2024): e13705, https://doi.org/10.1111/cpr.13705.

In the originally published article, Mu Yang's affiliations were incorrectly listed as “Mu Yang^1,2^”. The correct affiliation is shown below:

Mu Yang^2^

^2^Centre for Translational Research in Cancer, Sichuan Cancer Hospital and Institute, Sichuan Cancer Center, School of Medicine, University of Electronic Science and Technology of China, Chengdu, China

We apologise for this error.

